# Application of Machine Learning to Automated Analysis of Cerebral Edema in Large Cohorts of Ischemic Stroke Patients

**DOI:** 10.3389/fneur.2018.00687

**Published:** 2018-08-21

**Authors:** Rajat Dhar, Yasheng Chen, Hongyu An, Jin-Moo Lee

**Affiliations:** ^1^Division of Neurocritical Care, Department of Neurology, Washington University in St. Louis, St. Louis, MO, United States; ^2^Division of Cerebrovascular Diseases, Department of Neurology, Washington University in St. Louis, St. Louis, MO, United States; ^3^Department of Radiology, Washington University in St. Louis, St. Louis, MO, United States

**Keywords:** ischemic stroke, machine learning, cerebral edema, image analysis and processing, CT scan, CSF volume, GEE

## Abstract

Cerebral edema contributes to neurological deterioration and death after hemispheric stroke but there remains no effective means of preventing or accurately predicting its occurrence. Big data approaches may provide insights into the biologic variability and genetic contributions to severity and time course of cerebral edema. These methods require quantitative analyses of edema severity across large cohorts of stroke patients. We have proposed that changes in cerebrospinal fluid (CSF) volume over time may represent a sensitive and dynamic marker of edema progression that can be measured from routinely available CT scans. To facilitate and scale up such approaches we have created a machine learning algorithm capable of segmenting and measuring CSF volume from serial CT scans of stroke patients. We now present results of our preliminary processing pipeline that was able to efficiently extract CSF volumetrics from an initial cohort of 155 subjects enrolled in a prospective longitudinal stroke study. We demonstrate a high degree of reproducibility in total cranial volume registration between scans (*R* = 0.982) as well as a strong correlation of baseline CSF volume and patient age (as a surrogate of brain atrophy, *R* = 0.725). Reduction in CSF volume from baseline to final CT was correlated with infarct volume (*R* = 0.715) and degree of midline shift (quadratic model, *p* < 2.2 × 10^−16^). We utilized generalized estimating equations (GEE) to model CSF volumes over time (using linear and quadratic terms), adjusting for age. This model demonstrated that CSF volume decreases over time (*p* < 2.2 × 10^−13^) and is lower in those with cerebral edema (*p* = 0.0004). We are now fully automating this pipeline to allow rapid analysis of even larger cohorts of stroke patients from multiple sites using an XNAT (eXtensible Neuroimaging Archive Toolkit) platform. Data on kinetics of edema across thousands of patients will facilitate precision approaches to prediction of malignant edema as well as modeling of variability and further understanding of genetic variants that influence edema severity.

## Introduction

Over 10 million persons suffer a stroke each year worldwide (1). Most of these patients have at least one brain imaging study performed during their acute hospitalization, primarily for diagnostic purposes on presentation (2). Follow-up scans are often obtained to evaluate the size of infarction, degree of cerebral edema, as well as exclude the development of hemorrhagic transformation (3). Computed tomography (CT) is the most frequently employed modality for acute stroke imaging due to its widespread availability, lower cost, and greater speed of scanning, especially important in acutely unstable patients where “time is brain” (4). Although conventional CT does not have the ability of magnetic resonance imaging (MRI) to detect hyper-acute stroke, its ability to track progression of infarction and edema after stroke are comparable while affording greater temporal resolution with serial imaging (5). This practice means that there is a massive global imaging dataset of stroke patients with information on stroke location, infarct size, development of edema, and hemorrhagic transformation. While these parameters can be assessed by human raters, such evaluation is not scalable when leveraging imaging data from thousands of patients.

Cerebral edema develops around regions of brain infarction within the first week after stroke. This pathologic increase in brain water and hemispheric volume can lead to mass effect and is the major cause of death and neurological worsening after stroke (6). Development of edema is usually heralded by abrupt mental status worsening 2 days or more after admission, when herniation and midline shift have already developed (7). However, this process actually begins in the first hours after stroke and evolves continually and progressively over the first few days. At first decreases in blood and cerebrospinal fluid (CSF) compartments within the cranial compartment compensate for this increase in brain volume. However, once this has been exhausted, decompensation with worsening rapidly follows. Current measures of edema such as midline shift (MLS) or neurological deterioration capture only this decompensated state and not the critical early stages of edema before worsening. Further, assessing edema utilizing only MLS neglects the full spectrum of edema, including those with increased brain volume who never develop MLS. Measures of lesion volume either requires MRI (not feasible in all stroke patients) or can be estimated using CT; however, hypodensity on CT may be subtle early on and represents a variable combination of infarct plus edema. It is only the latter component that contributes to swelling and risk of herniation, and so lesion volume (even on MRI) only partially predicts risk of herniation (8).

We have proposed a sensitive quantitative metric of edema severity that can be extracted from CT imaging at variable time points after stroke (9). This leverages the reciprocal biologic relationship between increase in brain volume due to swelling and proportional decrease in CSF volume as compensation. CSF is pushed out of hemispheric sulci, cerebral ventricles, and the basal cisterns as edema develops in the hours and days after stroke. The reduction in CSF volume precedes the development of midline shift and clinical worsening due to edema. We demonstrated that the volume of CSF displaced up to the time of maximal edema closely correlated with extent of midline shift.

We have also developed an automated algorithm to segment CSF from CT scans of stroke patients (10). This critical step employed random forest-based machine learning (ML) trained on manually delineated scans. Features integrated into the ML platform include Haar-like patterns of pixels. This supervised learning approach was able to rapidly and reliably measure CSF volume on serial CT scans from two sites in our preliminary testing, performing significantly better than simple threshold-based models for CSF segmentation which were confounded by density of infarction mimicking CSF. Correlations of automated CSF volumes to ground-truth values exceeded 0.95, with volumes that closely approximated actual CSF values after active contour refinement. This automated approach facilitates the translation of this metric to studies evaluating edema in large numbers of stroke patients. Exploring the variability in quantifiable edema severity between patients will not only unlock opportunities for precise prediction of malignant edema at earlier time points but also provide the basis for understanding the genetic basis of cerebral edema. Such studies require thousands of stroke patients with serial imaging to undergo CSF-based edema measurement. We now present a proof-of-principle application of a processing algorithm capable of handling large datasets of CT scans and extracting CSF volumes for such analyses.

## Materials and methods

### Subjects and data collection

**Figure 1:**
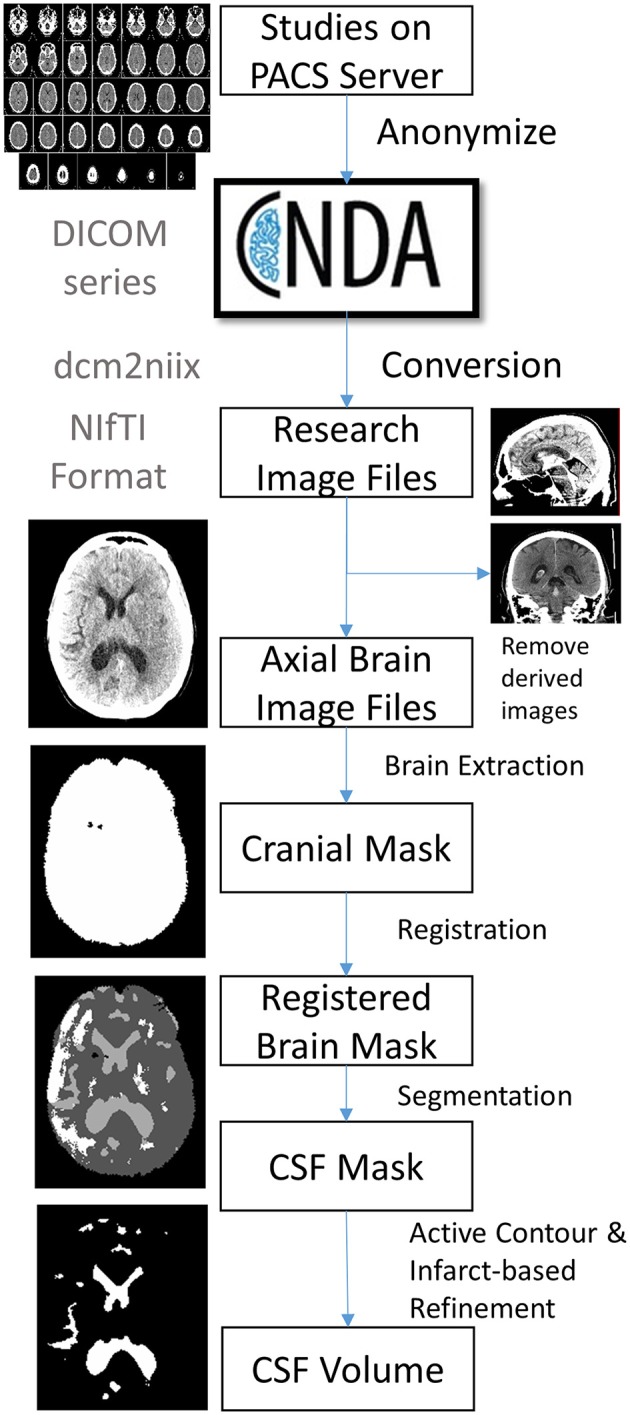
Outline of image processing pipeline to analyze CSF volumes from large cohorts of stroke patients.

Patients with a diagnosis of ischemic stroke who were admitted to Barnes-Jewish Hospital were screened for enrollment into the Genetics of Neurological Instability after Ischemic Stroke (GENISIS) study if they presented within 6 h of symptom onset. Subjects provided informed consent for data collection, including acute stroke imaging. Clinical data collected included age and NIHSS at baseline. All head CT imaging performed on subjects enrolled between 2009 and 2014 was then extracted from the clinical radiology server. We included only those with at least one follow-up scan performed during their hospitalization. Figure 1 shows the steps involved in a processing pipeline capable of uploading, evaluating, processing, and extracting CSF volumes from these scans. All scans (including baseline CT on presentation and each follow-up scan available) were uploaded from the hospital's Picture Archiving and Communication System (PACS) server to Central Neuroimaging Data Archive (CNDA), where they were stored in Digital Imaging and Communications in Medicine (DICOM) format (11). All studies were de-identified during the upload process using a standard algorithm integrated into the upload pipeline. FU scans were reviewed for presence of visible infarct as well as graded for degree of cerebral edema (CED grade 0, no infarct visible; 1, focal swelling up to 1/3 of cerebral hemisphere; 2, focal swelling of >1/3 of cerebral hemisphere; 3, swelling with midline shift) (12).

### DICOM conversion

DICOM images were converted to NIfTI (Neuroimaging Informatics Technology Initiative) format in bulk using the *dcm2niix* software. Multiple DICOM-encoded brain slices from a single scanner sequence were compiled into a single 3-dimensional NIfTI file. The header of the newly created NIfTI file also stores the image dimensions (e.g., ~512 × 512 × 32) and pixel dimensions (e.g., ~0.42 × 0.42 × 5-mm). The conversion also labels the resulting file using the subject identifier (assigned during upload) plus date and time of each scan (extracted from the DICOM metadata). Due to inconsistency in storing slice thickness in CT metadata, conversion extracts pixel height not from slice thickness and/or spacing between slices (reported inconsistently in metadata) but by calculating the actual distance between two consecutive slices. Conversion of CT images poses additional unique complexities: images are often acquired with slice axis oblique to the scanning table (13). This *gantry tilt* would result in a skewed 3D stack of images if this is not resolved using trigonometry and resampling (as is performed during conversion). This resampling to a consistent plane is also important for accurate co-registration of scans within a given patient. Some CT series may also be acquired with varying slice thicknesses, typically with thinner slices in the posterior fossa. Such inconsistency cannot be handled by the NIfTI format, which requires uniform slice thickness when storing imaging data. The conversion algorithm recognizes such variable inter-slice distances and interpolates to a uniform thickness in the resulting NIfTI file. We also store additional metadata not captured in the NIfTI header (such as scanner, protocol, method of conversion) in a brain imaging data structure (BIDS) accessory file (14).

### Image selection

Each patient often has multiple series performed as part of a single session. Derived images were excluded automatically from conversion using the “–*i y*” switch in dcm2niix. However, selection of axial brain images required some manual review of converted NIfTI files to exclude bone windows and additional series that were not analyzed (e.g., angiographic images).

### Infarct review

Each follow-up scan was also manually reviewed for presence and location of visible infarcts as well as presence and degree of midline shift (at level of the septum pellucidum). Visible stroke-related hypodensities were outlined in MRICro and saved as image masks. Infarct location was categorized as cortical, subcortical, both cortical and subcortical, lacunar (subcortical with diameter < 15 mm), or other.

### Brain extraction and perimeter registration

Further anonymization of images was ensured by removal of all structures external to the cranial cavity (i.e., skull stripping). This was accomplished by k-means clustering of pixel intensities to segregate brain, skull, and all external pixels. Skull and external regions were then excluded to yield a mask of just the intracranial contents. This image was then registered to a brain template that consisted of 15 brain images of stroke patients with manually outlined cranial perimeter to include all supra-tentorial structures as well as basal cisterns, but specifically excluding portions of the posterior fossa (e.g., cerebellum) on the same slices. Each subjects baseline brain scan was registered to each of these atlas brains using the Advanced Normalized Toolkit (ANTS) and pixels were included if they matched to the atlas masks in over half of the template scans. This registered baseline scan was then registered to each follow-up scan and non-matching brain regions were excluded.

### CSF segmentation

**Figure 2:**
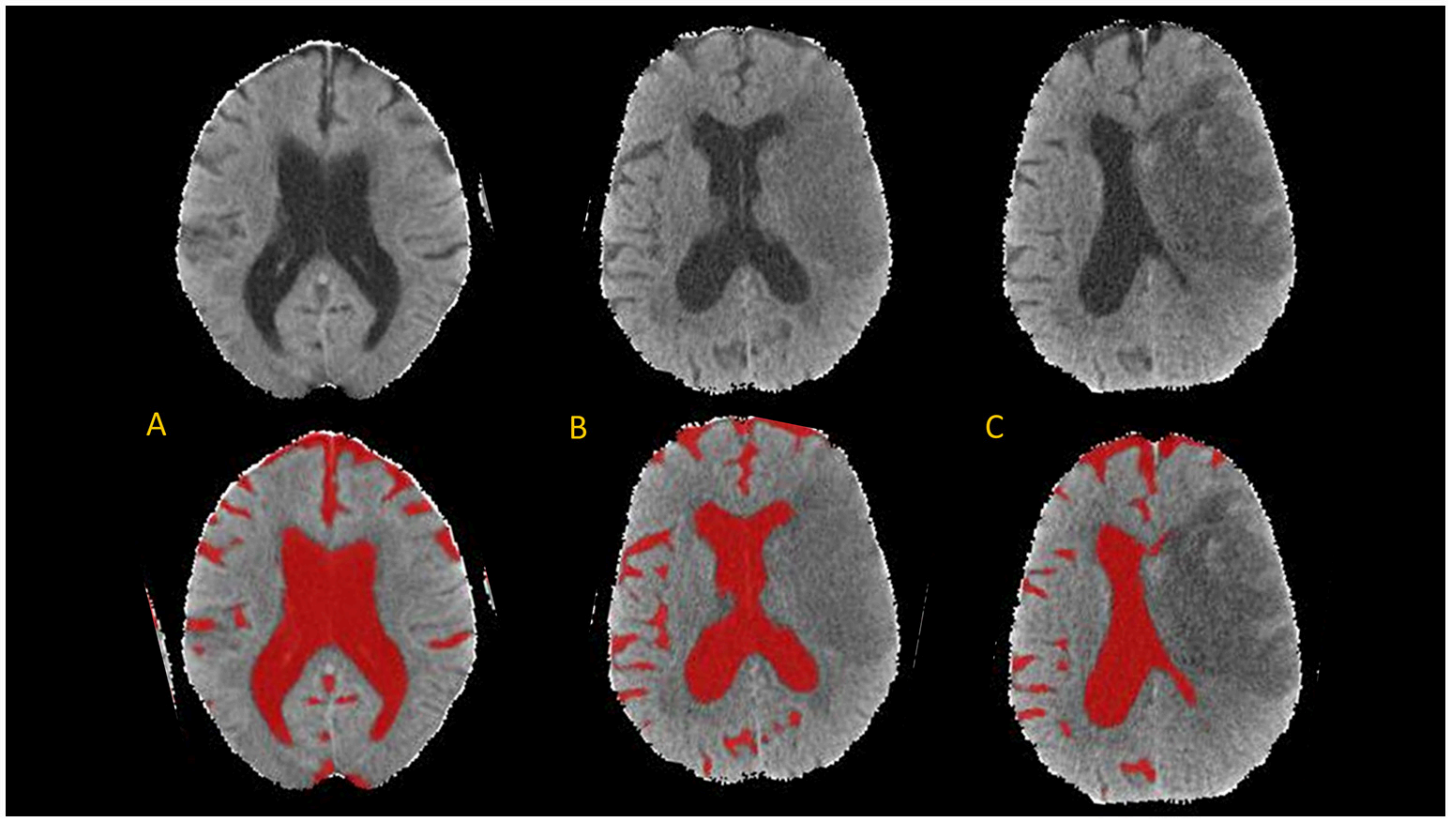
Axial brain slices from head CT (top) and results of CSF segmentation from a 82-year old woman with initial NIHSS of 18. Baseline CT **(A)** was performed within 1 h of stroke onset (CSF volume 224 ml). First follow-up CT **(B)** was performed at 20-h (CSF volume 150 ml) and second follow-up CT **(C)** at 110-h (CSF volume 105 ml).

The brain mask was then segmented using the CSF classifier that we previously trained using random forest machine learning (10). This segmentation was then refined using an active contour method and cleaned using a manually drawn mask of the infarct hypodensity (if present on follow-up scans). Results are summarized in JPEG snapshots of the resulting CSF mask overlaid onto the CT images for manual review of segmentation accuracy on serial scans (see Figure 2 for results of CSF segmentation in one representative subject).

### Volumetric analyses

The number of pixels in each compartment (intracranial compartment, CSF, infarct) is extracted from each image mask. This is then converted into volume using the pixel dimensions in the image header. Results from each scan are compiled into an exportable data file. This was analyzed in R (R: a language and environment for statistical computing). CSF and infarct volumes were analyzed in milliliters (ml) as well as a proportion of total cranial volume (%). The maximum change in CSF volume was calculated using the lowest measured volume as a percentage of the baseline volume.

### Dynamic CSF volumetric modeling

Generalized estimating equation (GEE) was used to model the temporal CSF volume changes using the multiple CT scans from this patient cohort. In this study, due to an irregular time interval between the scans from different subjects, we employed a Markov working correlation structure, $corr\left(y_{i,j},y_{i,k}\right)=a^{\left|t_{i,j}-t_{i,k}\right|}$, where *y*_*i,j*_ and *y*_*i,k*_ are CSF volumes of patient *i* at *t*_*ij*_ and *t*_*ik*_. Besides its capability to model irregular time interval between the scans, this working correlation structure also takes the assumption that the correlation between the measurements from the same subject weakens with an increased time interval (0 < *a* < 1) (15). In this study, the model we employed for statistical inference include age, time from stroke onset (T), and a dichotomized cerebral edema grade (CED grade 3 vs. grade 0, 1, 2), which is given as *E*(*y*_*i,j*_) = *b*_0_ + *b*_1_*t_*i,j*_ + *b*_2_*t_i,j_*t_*i,j*_ + *b*_3_*age_*i*_ + *b*_4_*ced_*i*_. These coefficient (b0~b4 and a) are calculated through a two stage solutions. *P*-values were computed with a robust covariance structure.

## Results

The cohort included 155 subjects, whose demographics are shown in Table 1. Registration failed in two subjects, who were excluded from segmentation and analysis. This left a total of 397 scans analyzed for cranial cavity and CSF volumes. Median time from stroke onset to first scan was just over 1 h (IQR 0.8–2.4 h) while time from baseline to first follow-up scan was a median of 21 h (IQR 6–42 h); 55 subjects had three or more scans performed serially after stroke. One hundred (66%) had one or more scans performed at least 24 h after stroke onset. In one case the only FU scan was over 1 week after stroke; this subject was excluded. The majority of infarcts were cortical or both cortical and subcortical. In those with at least 24-h follow-up, median volume of visible infarct-related hypodensity was 73 ml (IQR 5–203). Median volume was 22 ml for subcortical infarcts, 49.6 ml for cortical infarcts, and 219 ml for infarcts affecting both cortical and subcortical regions.

**Table 1:** Cohort of 155 stroke subjects with baseline and follow-up CT scans.

**Variable**	
Age	67 ± 14 years
Gender, male	82 (53%)
NIHSS	11 (IQR 6–16)
Time from stroke onset to first CT	1.25 h (IQR 0.8–2.4)
Number with 2/3/4/5 serial scans	153/55/24/14
**Infarct location:**	
Cortical only	24
Subcortical only	8
Both cortical and subcortical	42
Lacune	7
No infarct seen	12
Unable to assess (no scan beyond 24 h)	56
CED grade^*^0/1/2/3	17/34/16/32

* *In those with imaging at least at 24 h or beyond*.

**Figure 3:**
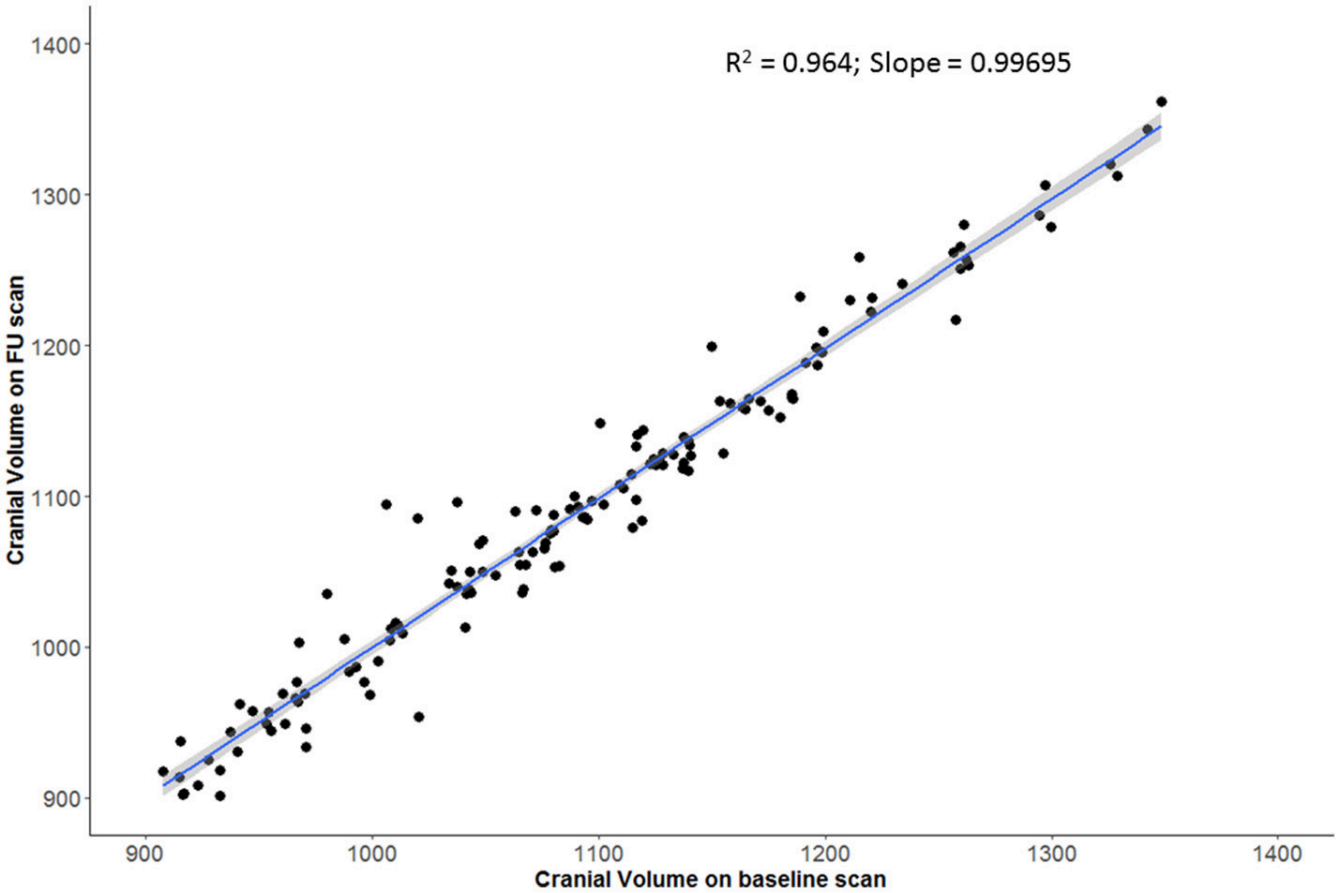
Strong agreement of registered cranial volumes on baseline and follow-up scans.

**Figure 4:**
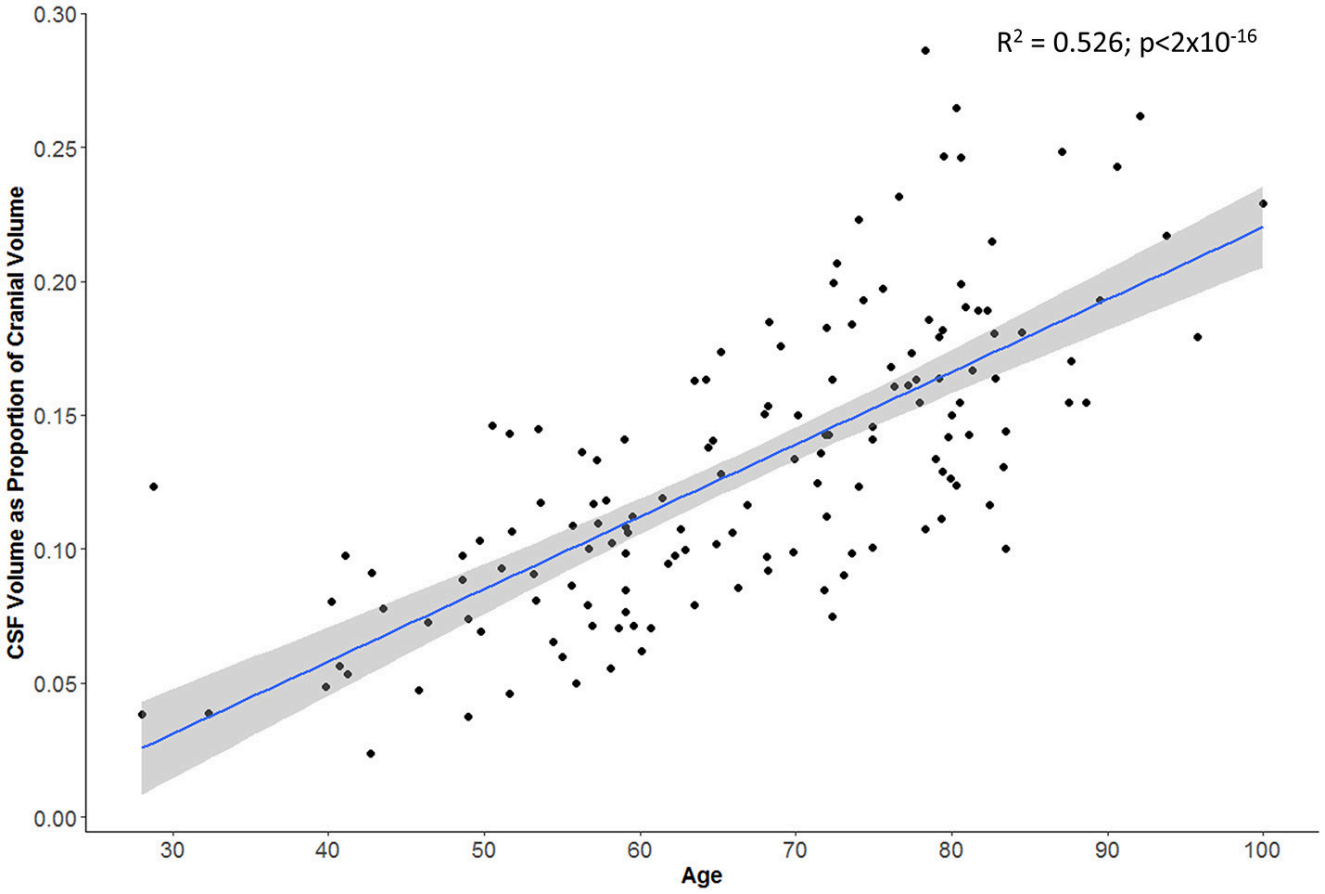
Subject's age correlates with proportion of cranial cavity comprised by CSF on baseline head CT (gray zone represents 95% confidence interval for predictions from the linear regression model).

Swelling with midline shift (i.e., CED grade 3) was demonstrated in 32 (32%) of those with scans beyond 24 h. Median MLS was 6.5 mm in this subgroup (IQR 4.0–9.5) compared to 0 in other CED grades. Registration was able to extract a consistent cranial mask across serial scans; we demonstrated a strong correlation between baseline and FU cranial volumes (*r* = 0.98, *p* < 2 × 10^−16^; Figure 3). There was also a good correlation between baseline CSF volume (as percent of cranial volume) and age of patient (*r* = 0.74, *p* < 2 × 10^−16^; Figure 4).

**Figure 5:**
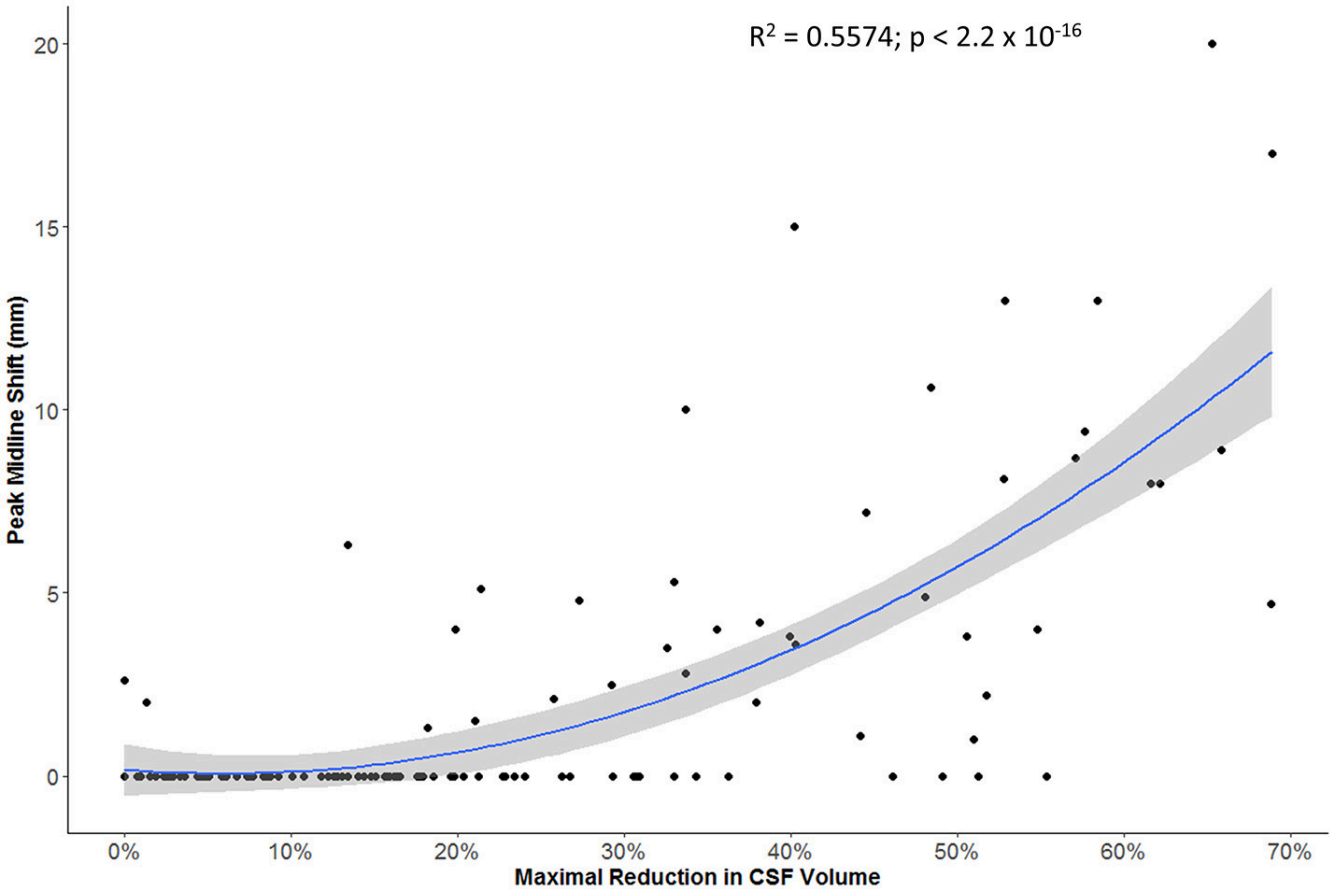
Relationship between maximal reduction in CSF volume (relative to baseline) and peak degree of midline shift (gray zone represents 95% confidence interval for predictions from the quadratic regression model).

**Figure 6:**
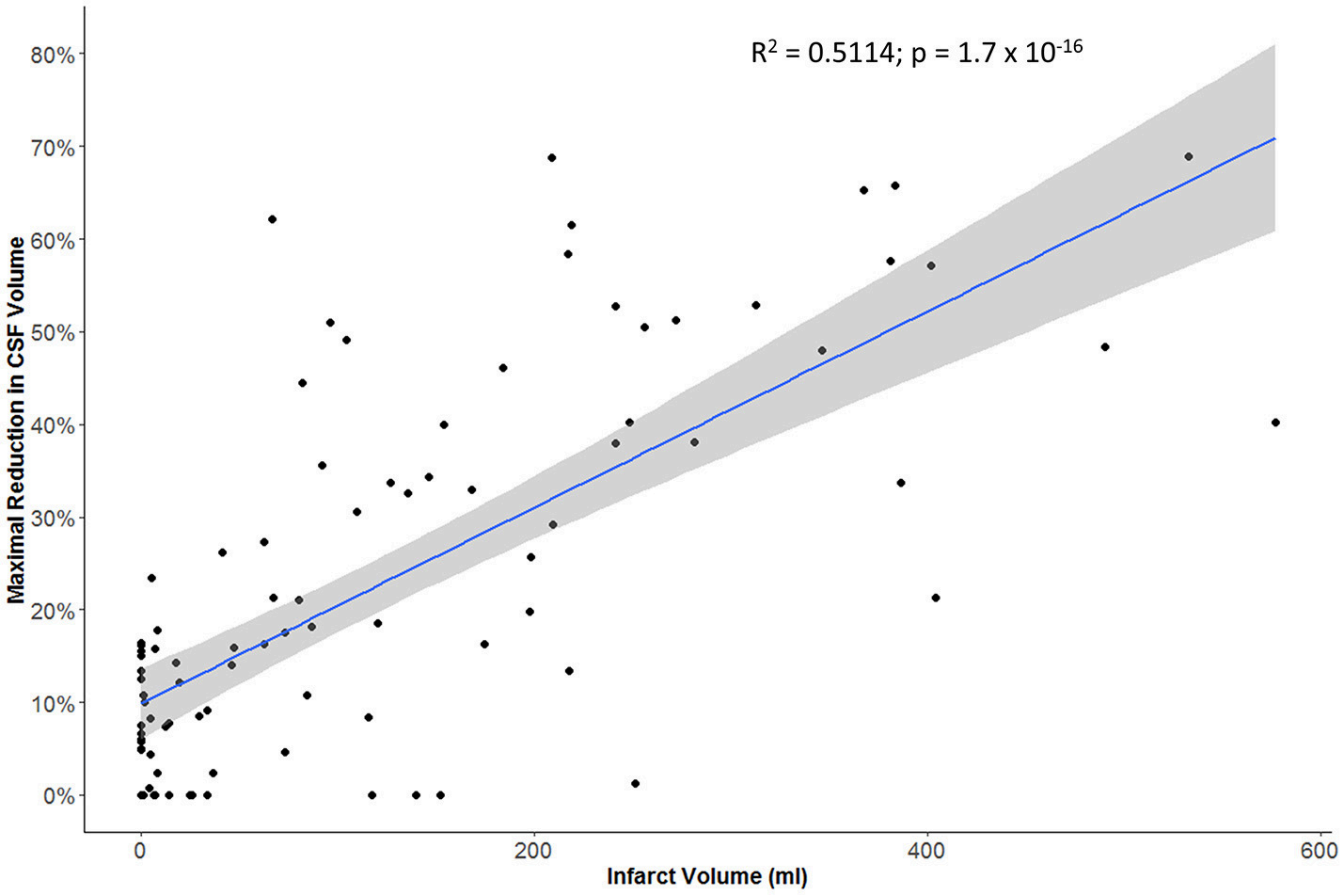
Relationship between infarct volume (based on largest hypodensity measured from available head CT scans) and maximal reduction in CSF volume.

**Figure 7:**
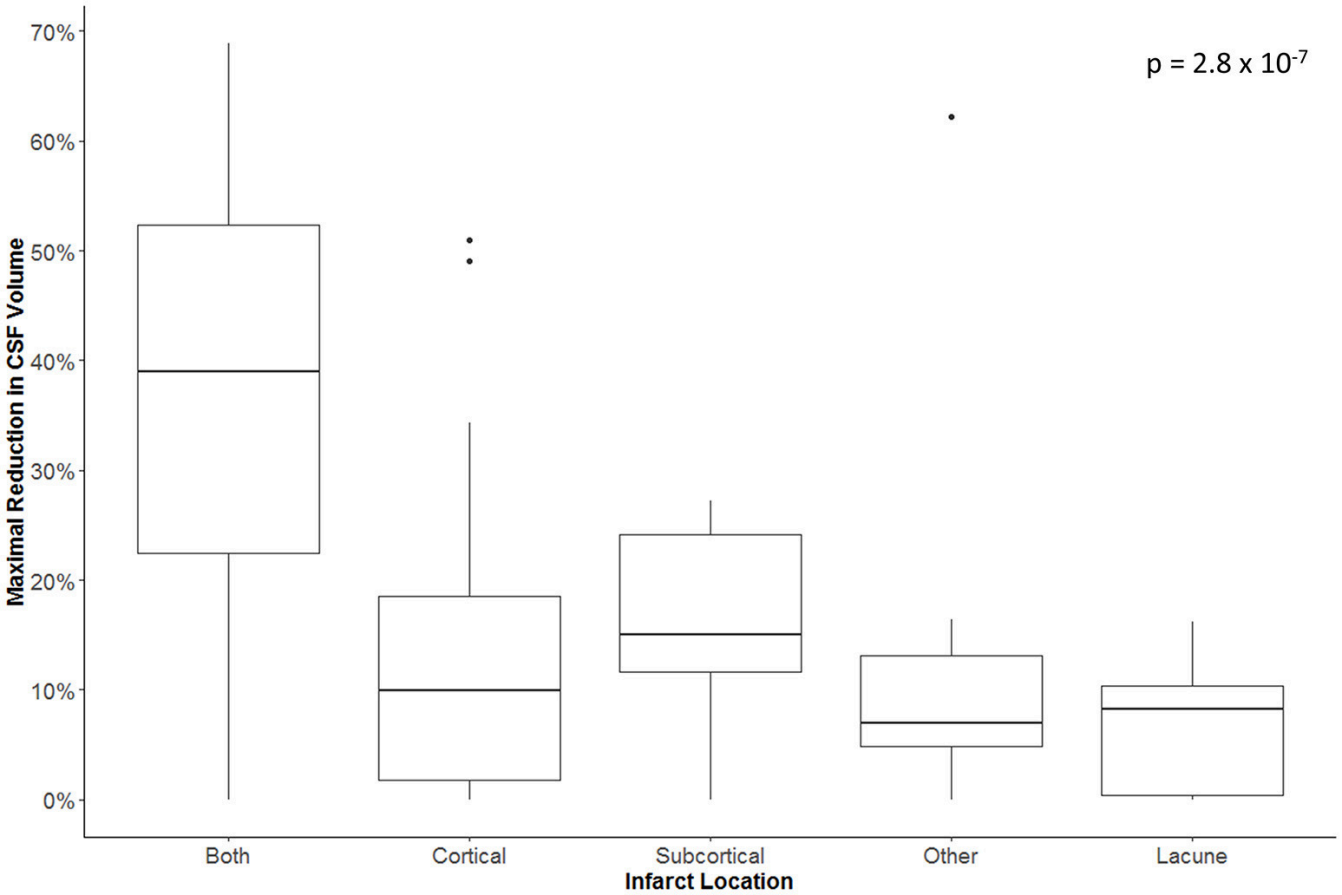
Maximal reduction in CSF volume (as percentage of baseline) in relation to infarct location.

The maximal reduction in CSF volume (as percentage of baseline) was associated with degree of midline shift developing (Figure 5). In fact, there appeared to be a non-linear (quadratic) relationship, whereby minimal midline shift developed despite a mild-moderate CSF volume loss. Beyond the point at which 30–40% of the total baseline CSF had been lost, it appears that midline shift rapidly develops. Peak CSF volume loss was also correlated with infarct volume in a linear fashion (Figure 6) and was significantly greater in those stroke patients with infarcts affecting both cortical and subcortical structures and minimal in those with lacunar infarcts (Figure 7).

**Figure 8:**
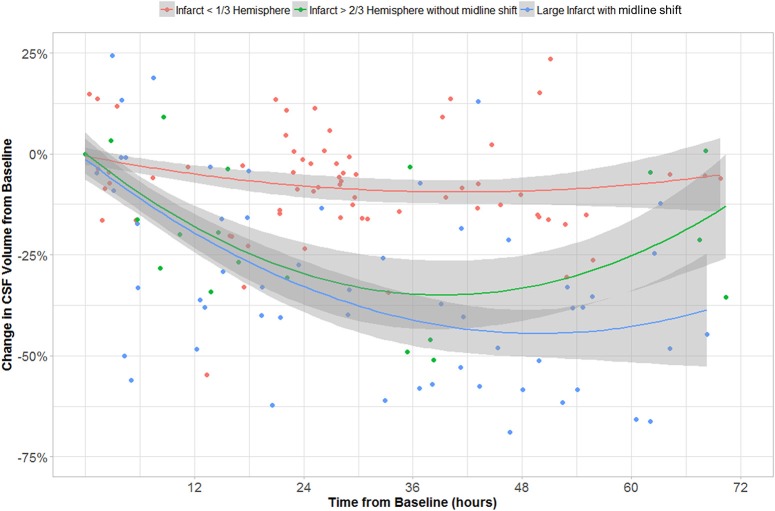
CSF volume (as percentage of volume on baseline CT) over time in groups with CED grades 0–1, 2, and 3.

In the GEE model, we found that CSF volume was independently affected by all three variables: age, time from stroke onset and CED grade. CSF volume increases with age (*b*_1_ = 3.01 cc/year, *p* < 10^−16^) and is lower in those with CED grade 3 (*b*_3_ = −32.57 cc, *p* = 4 × 10^−4^). CSF volume also decreased over time (−22 cc/day, *p* = 2 × 10^−13^) but there was also a second-order quadratic time factor significant in CSF evolution (*p* = 6 × 10^−10^). The evolution of CSF volume over time in CED grades is shown in Figure 8.

## Discussion

Here we present the initial results of a machine learning-based pipeline to analyze large numbers of serial CT brain images in order to quantify the progression of cerebral edema after ischemic stroke. We applied our random forest-based segmentation algorithm within a broader image processing pipeline to measure CSF volumes in almost 400 CT scans, with failure of scan registration in only two of over 150 subjects with a variety of stroke locations and volumes. We are now working to refine our registration parameters to deal with these rare failures, including addition of shrink factors, smoothing parameters, and affine registration (16). In the remainder cranial registration was robust, with tight correlation of volumes between baseline and repeat images. We also demonstrated a clear relationship between proportion of the cranium comprising CSF (as a surrogate for brain atrophy) and patient age (17).

More importantly, we further demonstrated that our metric of CSF volume reduction is a strong marker not only of stroke volume but of the eventual development of midline shift. There was more CSF volume loss in those with larger infarcts affecting both cortical and subcortical structures. However, it appears that midline shift only develops once some degree of compensation afforded by CSF loss has been exhausted. Beyond this threshold, midline shift rapidly develops, as illustrated by our quadratic modeling.

Furthermore, we used longitudinal GEE modeling to demonstrate that CSF volume generally decreased over time after stroke. Even adjusting for age and time from baseline CT, we confirmed that those with significant CED had greater reductions in CSF volume than those without CED. CSF volumes do not change appreciably over time in those with small infarcts (Figure 8) with while those with larger infarcts (CED grades 2 and 3) appear to exhibit gradual but progressive reductions in CSF volumes of between 25 and 50% relative to baseline. Furthermore, those with CED grade 3 (who, by definition, ultimately develop MLS) seem to manifest a continued downward trajectory between 24 and 48 h after stroke. This group appears to reach an asymptote of maximal CSF reduction of about half baseline volume by 48 h. This volume reduction would represent approximately one hemisphere of CSF, appropriate to a process that is likely to produce edema predominantly involving the ipsilateral hemisphere. As our analysis relating MLS with CSF volume loss suggests, there is potential for greater decompensation (with development of MLS) once this proportion of CSF volume has been exhausted. As we accumulate more volumetric data across more stroke patients, we plan to perform more sophisticated analyses that evaluate the interaction of edema severity with rate of CSF volume reduction, incorporating and modeling the effect of further covariates such as NIHSS.

This study provides proof-of-principle that we can automate brain imaging data analysis and obtain meaningful volumetric data on large cohorts of stroke patients. Such an approach, leveraging routinely obtained clinical imaging data or imaging obtained in clinical trials to advance the science of stroke is the pathway to realizing the potential of big data in brain imaging (18). One notable challenge of sharing brain imaging data is ensuring anonymization. In our pipeline this is accomplished by both robust de-identification of DICOM metadata prior to scan transmission to our centralized repository as well as skull stripping during brain extraction and registration. This latter process has also been accomplished previously using similar methods (19).

While this study demonstrates the feasibility of an imaging pipeline to deal with large volumes of CT data, there are a number of refinements required before it can manage big imaging data from large multi-site repositories. In this preliminary test application, we only utilized data from a single site with existing upload capabilities from PACS to our analysis server. In future we will leverage the existing resources of CNDA to import and archive scans from multiple sites. This imposes other challenges to imaging harmonization as scans are obtained with various protocols under varying sequence names and even in disparate languages. We are currently developing a convolutional neural network (CNN) approach to intelligently but automatically select the appropriate scan from a number of CT series performed concurrently. Subsequent steps in processing such as brain registration and segmentation also need to be automated and we are working on a Docker container-based approach to integrating processing modules (20). We are also working to provide internal quality control checks and means of project-level data visualization to further refine the processing. A further challenge to full automation is the need for manual delineation of infarct hypodensity. We are now developing a CNN-based method of segmenting stroke lesions from serial CT scans (21). Such refinements will be key to successfully scaling up these processes to thousands of CT scans and realizing the potential of big data in stroke. With respects to cerebral edema, this will allow us to precisely predict the course of individual patients from early CSF changes while simultaneously utilizing this imaging-based endophenotype (rate of edema formation) as the basis for powerful genetic studies to develop new targeted therapies to prevent edema.

## Ethics statement

The study protocol was approved the Human Research Protection Office at Washington University in St. Louis. All subjects gave written informed consent in accordance with the Declaration of Helsinki.

## Author contributions

RD drafted the initial manuscript and collected the clinical data. YC performed the image processing, statistical analyses, and revised the manuscript. HA and J-ML supervised this project, reviewed the manuscript, and made critical revisions.

### Conflict of interest statement

The authors declare that the research was conducted in the absence of any commercial or financial relationships that could be construed as a potential conflict of interest.
